# Exploration of a somatosensory interactive assessment tool for children with intellectual disabilities

**DOI:** 10.1002/pchj.759

**Published:** 2024-04-17

**Authors:** Feng Lu, Panpan Li, Fanlin Zeng

**Affiliations:** ^1^ School of Education Science Taizhou University Taizhou China; ^2^ Department of Special Education, Faculty of Education East China Normal University Shanghai China

**Keywords:** assessment, children with intellectual disabilities, somatosensory interactive technology, tool design

## Abstract

Based on the functional assessment concept and embodied assessment requirements, the present study aimed to design and develop an assessment tool for children with intellectual disabilities with the help of somatosensory interactive (SI) technology. The sample in this study consisted of 73 children with intellectual disabilities and 70 children with typical development. Data were collected through three SI tasks, four traditional executive function tasks, and user experience interviews to analyse the effectiveness of the SI assessment tool. The results showed that the SI assessment tool had good scale validity, discriminant validity, and the ability to identify intellectual disabilities. Children preferred SI tasks and showed higher involvement and more positive emotions. The SI tool with three SI tasks is a more scientific, effective, and advanced tool for assessing children with intellectual disabilities.

## INTRODUCTION

Intellectual disability (ID) is a neurodevelopmental disorder that children with ID have intellectual disability and social adjustment dysfunction at the developmental stage (Zeilinger et al., 2022). According to official data released by the China Disabled Persons' Federation (CDPF), based on the results of the sixth national population census and the second national sampling survey of disabled people, it is estimated that individuals with ID in China make up approximately 6%–7% of the total number of disabled people, representing 5–6 million people (CDPF, 2021). People with ID therefore represent a huge group with special needs (Mckenzie et al., 2023; Patel et al., 2023). With the progress of social concepts and technology, we should be able to provide them with more scientific and efficient help (Blocksidge et al., 2023). Assessment is the starting point and foundation of this approach.

Operant psychometry is widely used to assess the intelligence of children with ID, using measures of attention and executive function (Bexkens et al., 2013; Cho et al., 2013; Park et al., 2011). This method, combined with scale assessment, can provide data support for diagnosing children with ID, but has many shortcomings, such as boring tasks or difficult operations that children cannot perform (Erostarbe‐Pérez et al., 2022; Fidler & Lanfranchi, 2022). In addition, the highly experimental environment means that the measurement results are different from the actual abilities of the children in their living environments, and the ecological validity of the measurement is low (Barclay et al., 2018; Li et al., 2017). Therefore, on the basis of traditional operational psychometric principles, this study introduces the technology of somatosensory interaction (SI) to form an assessment tool suitable for studying the characteristics of children with ID, involving tasks that are more fun and that have greater ecological validity.

SI technology (SIT) is a digital technology based on virtual reality that is gradually being applied in many fields in China. SIT comprises hardware and software. First, the hardware captures information on the individual's body, such as movement, facial expression, and voice; then, the captured information is analysed and judged by data analysis software, and output information is provided. After the introduction of SIT, researchers have tried to apply it in various fields, such as medical rehabilitation (Zhang et al., 2023) and education and training (Chen et al., 2023), and suggested that SIT could effectively improve the effects of training and education (Golden et al., 2022; Guo et al., 2022). SIT has also been applied in interventions for children with special needs. For example, Xu and Zhu (2016) showed that 8 weeks of SI games could effectively improve the physiological function, daily living ability, and communication ability of children with ID. Alqithami et al. (2019) showed that SI games could improve attention among children with attention‐deficit/hyperactivity disorder (ADHD) and were more efficient than traditional methods. Zhang et al. (2023) proved that SI games could improve the intelligence and motor development of children with cerebral palsy.

The value of the new assessment tool lies not only in the application of state‐of‐the‐art technology but also in its use of new concepts and theories related to the assessment and cognitive development of children with ID. First, the assessment of children with ID should not be limited to traditional intelligence measurement, it is also important to evaluate adaptability and the required level of external support in their interaction with the environment (Schalock et al., 2018) to realize the functional assessment of children with ID (Luckasson & Schalock, 2013). Second, the increasing popularity of embodied cognitive theory has promoted reflection on cognitive ability assessment and provided a new theoretical perspective. This theory emphasizes that cognitive processes are based on the body and the environment (Ye et al., 2018). The assessment of cognitive ability should not be limited to brain cognition but should also obtain information on the interaction between the body and the environment to realize the integrated assessment of the mind, body and environment. Studies on movement training of children with ID showed that training focusing on environmental feedback was more effective than training focusing solely on movement feedback (Chiviacowsky et al., 2013). As environmental intervention is an important aspect of intervention for children with ID, the assessment of children with ID should fully consider environmental settings. The development of SI technology allows the design of tools based on the concept of functional assessment that meet the requirements of embodied assessment.

The assessment of children with ID aims to diagnose intellectual disabilities and provide a basis for their classification. More importantly, assessment can help in the development of more targeted and individualized education plans (Don & O'Byrne, 2022; Qiu et al., 2016). Therefore, the development of an assessment tool must consider how to effectively identify and diagnose disabilities and how to provide additional information for later intervention and education. SI technology has already improved interventions for children with ID, and its application in the development of an assessment tool will likely promote a better connection with subsequent individualized interventions.

Based on the functional assessment concept of children with ID and the embodied cognition theory, the second generation of cognitive science, and with the help of SI technology, this study intended to design and develop a cognitive assessment tool for children with ID using the executive function assessment of children with ID as an example. When designing SI assessment tasks, the requirements of functional assessment and embodied cognition theory are met through task demands and digital environmental settings. Compared with traditional tasks that require only hand–eye coordination, SI tasks necessitate full‐body involvement. Participants are required to coordinate movements of their head, hands, feet, and torso to complete the task. The SI tasks are set in daily‐life scenarios or in scenarios enriched with diverse environmental elements, and the feedback provided during the tasks involves information from multiple sensory channels. These design choices are based on the theory of embodied cognition and will effectively enhance the ecological effectiveness of SI tasks. By testing the reliability and validity of the tool, analysing the sensitivity and specificity of the tasks in the diagnosis of children with ID, and evaluating the user experience and engagement of children, this study ensures the scientific, effective and advanced nature of the tool developed.

## MATERIALS AND METHODS

### Participants

The sample comprised 73 children with ID and 70 with typical development (TD). The children with ID were recruited from one special education school (19 children) and from regular classes in 11 ordinary schools (54 children) in Shanghai, China. The children with TD were recruited from two ordinary primary schools and one ordinary junior high school in Shanghai.

The participants were selected according to the IQ scores provided by the schools. The experimental group included students with mild intellectual disabilities, with IQs between 50 and 69, and students with moderate intellectual disabilities, with IQs between 35 and 49. Ultimately, the experimental group included 52 children with mild ID and 21 with moderate ID, ranging from 9 to 16 years old, with an average age of 12.08 ± 1.94 (*M* ± *SD*) years. All of the children were enrolled in grades 3 to 9. A total of 70 age‐ and sex‐matched TD children aged 6–16 years were selected to form the control group. All the children in the control group took the Raven intelligence test in their spare time, and their IQ was normal. None of the participants had vision, hearing, motor, emotional or behavioural disorders.

A total of 122 children participated in the user experience interviews, with valid data from 120 participants (63 with ID and 57 with TD). Test videos of 20 participants (10 with ID and 10 with TD) were randomly selected for participation analysis.

### Tools

The experimental equipment included a Microsoft Kinect1.0 SI device (Made in USA), a Lenovo laptop computer (Made in China), and a Canon camera (Made in Japan).

The executive function SI assessment tool for children with ID included three tasks: SI Go‐Nogo, SI Stop‐Signal, and SI Coding. The validity of the SI assessment tasks was tested using the traditional executive function measurement results of the Stroop color‐word test, trail‐making test, coding test, and digit span. SI tasks were designed based on the unique requirements of functional assessment and embodied cognition theory.

In the SI Go‐Nogo task, the numbers or letters in the Go‐Nogo experimental paradigm were changed to hamsters and rabbits for a more physical experience, and participants were asked to hit the hamsters and avoid hitting the rabbits. Right or wrong strokes provided auditory and visual feedback. The ratio of correct hits (SI‐A1) and the average response time of correct hits (SI‐A2) were the task indices.

The SI Stop‐Signal task applied the stop‐signal task paradigm to a driving scenario. A red light represented the stop signal, and a green light represented the reaction signal. When the participant made a fist, the car moved forwards, and when the participant opened their palm, the car stopped. Visual and auditory feedback was given when the participant stopped correctly, incorrectly or not in time. The ratio of correct stopping at the red light (SI‐B1) and the average response time of correct stopping at the red light (SI‐B2) were the indices of this task.

The SI Coding task presented the coding task in the Wechsler children's intelligence test in the form of hitting water drops. Numbers 1 to 5 corresponded to squares, circles, triangles, crosses, and pentagrams, respectively. Participants were required to select the corresponding number by rotating the barrel according to the shapes on the screen. Visual and auditory feedback was provided when participants responded. The indices were the number of correct hits (SI‐C1) and the average response time of correct hits (SI‐C2).

The criterion validity was judged by the measurement results of the Stroop color‐word test, trail‐making test, coding test, and digit span. These are classic tools with high reliability and validity, and are often used for the validity verification of new tools (Fidler & Lanfranchi, 2022; Lee et al., 2019). The Stroop color‐word test, for example, is often used as a valid measure in psychological assessment. Numerous studies have demonstrated the reliability and validity of the Stroop test across different populations and contexts (Aretsen et al., 2013; Scarpina & Tagini, 2017; Van der Elst et al., 2006), and the Stroop test has been used in clinical settings to assess cognitive functioning and diagnose conditions such as ADHD (Willcutt et al., 2005), learning disability (Leverett et al., 1999), etc.

The Stroop task presented the participants with two word lists with inconsistent meaning and font color and required them to complete two tasks of word reading and color reporting within 2 min. Correct word‐reading, color‐reporting (TM‐D1), and the rate of correct answers within the 2 min (TM‐D2) represented the participants' inhibition function. In the trail‐making task, participants were first required to connect the numbers 1 to 25 in order as soon as possible and then asked to cross‐connect the numbers 1 to 13 and letters A to L. The number of task errors (TM‐E1) and the time difference between the two tasks (TM‐E2) represents cognitive flexibility, and the completion time represents the processing speed (TM‐E3). The coding task required the participants to match symbols and numbers within 2 min, and the number of correctly drawn symbols represents the processing speed (TM‐F1). The digit span task required the participants to recite a number string read by the tester in forward and reverse order. Working memory capacity is represented by the maximum length of correctly recited numbers in forward and reverse order (TM‐G1, TM‐G2).

User experience preference was measured using structured interviews, including asking whether participants liked the motion‐sensing or traditional tasks, their desire to repeat the task, their degree of preference for the two tasks, and a brief description of their reasons. The user participation process was evaluated by watching videos from the two perspectives of engagement and emotional enthusiasm. The degree of engagement was evaluated by the frequency of participants' leaving the task during the experiment, and five grades evaluated emotional enthusiasm: 1 (*negative*), 2 (*slightly negative*), 3 (*neutral*), 4 (slightly positive), and 5 (positive).

### Procedures

All the tasks were completed in the school resource classroom or office during the participants' spare time, and the surrounding environment was kept quiet. Participants stood or sat on a chair, and the Kinect was placed approximately 1.5 m in front of them. The near‐infrared camera was adjusted to a position directly facing the participant's chest, and the computer was directly behind the Kinect. Participants completed the SI assessment tasks, traditional executive function measurements, and operational experience interviews in turn. All participants completed the test in a fixed order. First, the three tasks of SI Go‐Nogo, Stop‐Signal and Coding were completed, followed by the digit span, coding, trail‐making and Stroop tests. Finally, the task preference interview was completed. The participants were allowed to practice fully before the test and were formally tested after mastering and applying the rules. Considering the characteristics of the participants, especially children with ID, they were given a certain rest time between different tests to reduce the impact of fatigue, of about 1–2 min for children with TD and 2–3 min for children with ID. The basis for determining whether to start a new test was the emotional performance and verbal feedback of the participants. The test lasted 75 to 90 min. Ethical approval was obtained prior to the study from the Human Research Ethics Committee of East China Normal University, China. All participants and their guardians gave informed consent before the experiment.

## RESULTS

### Validity analysis of the assessment tool

#### Criterion validity

The correlation method was used to evaluate the criterion validity of the SI tasks, and correlation analysis was carried out between the indices of the SI tasks and the traditional measurement tasks. The results are shown in Table 1.

**TABLE 1:** Correlation analysis between SI indices and traditional measurement indices.

	SI‐A1	SI‐A2	SI‐B1	SI‐B2	SI‐C1	SI‐C2
TM‐D1 Inhibition	0.48**	−0.27**	0.39**	−0.51**	0.58**	−0.57**
TM‐D2 Inhibition	0.44**	−0.20*	0.32**	−0.39**	0.30**	−0.40**
TM‐E1 Flexibility	−0.51**	0.22**	−0.38**	0.46**	−0.48**	0.54**
TM‐E2 Flexibility	−0.42**	0.27**	−0.23**	0.29**	−0.46**	0.48**
TM‐E3 Speed	−0.54**	0.39**	−0.37**	0.43**	−0.59**	0.65**
TM‐F1 Speed	0.56**	−0.38**	0.47**	−0.51**	0.63**	−0.61**
TM‐G1 Memory	0.29**	−0.18*	0.27**	−0.30**	0.25**	−0.30**
TM‐G2 Memory	0.38**	−0.31**	0.30**	−0.33**	0.46**	−0.43**

*Note*: SI‐A1 and SI‐A2 represent the ratio of correct hits and response time in the SI Go‐Nogo task; SI‐B1 and SI‐B2 represent the ratio of correct stops and response time in the SI Stop‐Signal task; and SI‐C1 and SI‐C2 represent the total number of codes and response time in the SI Coding task, respectively. TM‐D1 and TM‐D2 represent the number of correct reads or reports and the accuracy rate in the traditional Stroop color word task; TM‐E1, TM‐E2, and TM‐E3 represent the number of errors in the trail‐making task, the completion time, and the time difference between task B and task A; TM‐F1 represents the total score of the Coding task. TM‐G1 and TM‐G2 represent forward and reverse digit spans, respectively.

** *p* < .01; * *p* < .05.

In the SI Go‐Nogo task, the ratio of correct hits (SI‐A1) showed a moderate correlation with the traditional indices, while the response time (SI‐A2) showed a weak correlation. The SI Stop‐Signal and Coding task indices were strongly correlated with the traditional indices. The indices of SI measurement were significantly correlated with the traditional indices, and the SI tool had good criterion validity.

The correlation coefficients were compared as described by meng1992 (Diedenhofen & Musch, 2015). The results showed that the correlation coefficients between SI‐A1 and processing speed (such as TM‐E3 and TM‐F1) and flexibility indices (TM‐E1) were significantly higher than those between SI‐A1 and memory capacity (TM‐G1 and TM‐G2) (*p*s < .05), but there was no significant difference between these coefficients and coefficients between SI‐A1 and inhibition function (TM‐D1 and TM‐D2). There was no significant difference between the correlation coefficients between SI‐A2 and each index. The correlation of inhibition, flexibility, and speed with SI‐A1 were significantly stronger than those with SI‐A2 (*p*s < .05), and the correlations of memory capacity with SI‐A1 and SI‐A2 showed no significant difference.

There was no obvious regularity in the correlation coefficients between SI‐B1 and the indices of traditional measurement tasks. The correlation between SI‐B2 and processing speed (TM‐E3, TM‐F1) was significantly stronger than that between SI‐B2 and memory capacity (TM‐G1, TM‐G2) (*p*s < .05).

The correlation coefficients of SI‐C1 and SI‐C2 with processing speed (TM‐E3, TM‐F1) were significantly higher than those with working memory capacity (TM‐G1) (*p*s < .05), and there was no significant difference with other correlation coefficients.

The correlation coefficients of SI‐C1 and SI‐C2 with inhibition, flexibility, and processing speed were significantly higher than those of SI‐A2, SI‐B1 and SI‐B2 (*p*s < .05). The correlation coefficients between SI‐A1 and inhibition, flexibility, and processing speed were significantly higher than those between SI‐A2 and SI‐B1 (*p*s < .05).

#### Discriminative validity analysis

The differences among TD children, mild‐ID children (Mi‐ID) and moderate‐ID children (Mo‐ID) for each index of SI tasks were compared to understand the differentiation degree of the SI task among different participants. The results are shown in Table 2.

**TABLE 2:** Analysis of variance of somatosensory task performance of different groups.

	TD	Mi‐ID	Mo‐ID	*F*	Bonferroni
SI‐A1	0.83 ± 0.11	0.63 ± 0.29	0.63 ± 0.23	16.30***	TD > (Mi‐ID, Mo‐ID)
SI‐A2	1055.45 ± 72.61	1084.13 ± 92.00	1158.31 ± 110.72	11.55***	TD < *^#^* Mi‐ID<Mo‐ID
SI‐B1	0.96 ± 0.07	0.86 ± 0.20	0.85 ± 0.21	8.34***	TD > (Mi‐ID, Mo‐ID)
SI‐B2	513.43 ± 76.67	610.13 ± 134.12	645.40 ± 106.85	19.25***	TD < (Mi‐ID, Mo‐ID)
SI‐C1	29.87 ± 6.31	23.33 ± 7.90	19.43 ± 5.69	24.76***	TD > Mi‐ID>Mo‐ID
SI‐C2	3169.70 ± 935.10	4868.69 ± 2258.99	5768.99 ± 2162.98	25.20***	TD < Mi‐ID<Mo‐ID

*Note*: The abbreviations in Table 2 have the same meanings as those in Table 1.

***p < .001; ^#^ .05 < *p* < .07.

SI‐A1 of the SI Go‐Nogo task could distinguish TD children from ID children, and SI‐A2 could effectively distinguish the three types of children. SI‐B1 and SI‐B2 of the SI Stop‐Signal task could distinguish TD children from ID children and were insensitive to the degree of intellectual disability. Both SI‐C1 and SI‐C2 of the SI Coding task could effectively distinguish the three types of children. The SI Coding task had the highest discrimination, followed by the Go‐Nogo and Stop‐Signal tasks.

### Recognition rate analysis

A receiver operator characteristic curve (ROC) was used to evaluate the recognition ability of the three SI assessment tasks for ID children. The results are shown in Table 3. According to the meaning of each index, the ROC test directions of SI‐A1, SI‐B1, and SI‐C1 were opposite to those of the other three indices.

**TABLE 3:** Recognition ability evaluation results of SI tasks.

	AUC	MYI	IMV	TPR	FPR	DCR	PPV	NPV
TG‐A1	0.76	0.49	0.769	0.69	0.80	0.74	0.78	0.71
TG‐A2	0.66	0.35	1113.05	0.51	0.84	0.67	0.77	0.62
TG‐B1	0.66	0.31	0.875	0.37	0.94	0.65	0.87	0.59
TG‐B2	0.76	0.43	549.12	0.71	0.71	0.71	0.72	0.70
TG‐C1	0.79	0.47	23.50	0.64	0.83	0.73	0.80	0.69
TG‐C2	0.79	0.47	4786.17	0.51	0.96	0.73	0.93	0.65

*Note*: AUC = area under ROC curve; DCR = diagnostic concordance rate, namely the rate of correct diagnosis; FPR = false‐positive rate, which is specificity; IMV = indicator measurement value, namely the indicator measurement value corresponding to the MYI; MYI = Maximum Youden Index, namely the maximum result of sensitivity plus specificity minus 1; NPV = negative predictive value, namely the rate of correct diagnosis in negative results; PPV = positive predictive value, namely the rate of correct diagnosis in positive results; TPR = true positive rate, which is the sensitivity.

**FIGURE 1:**
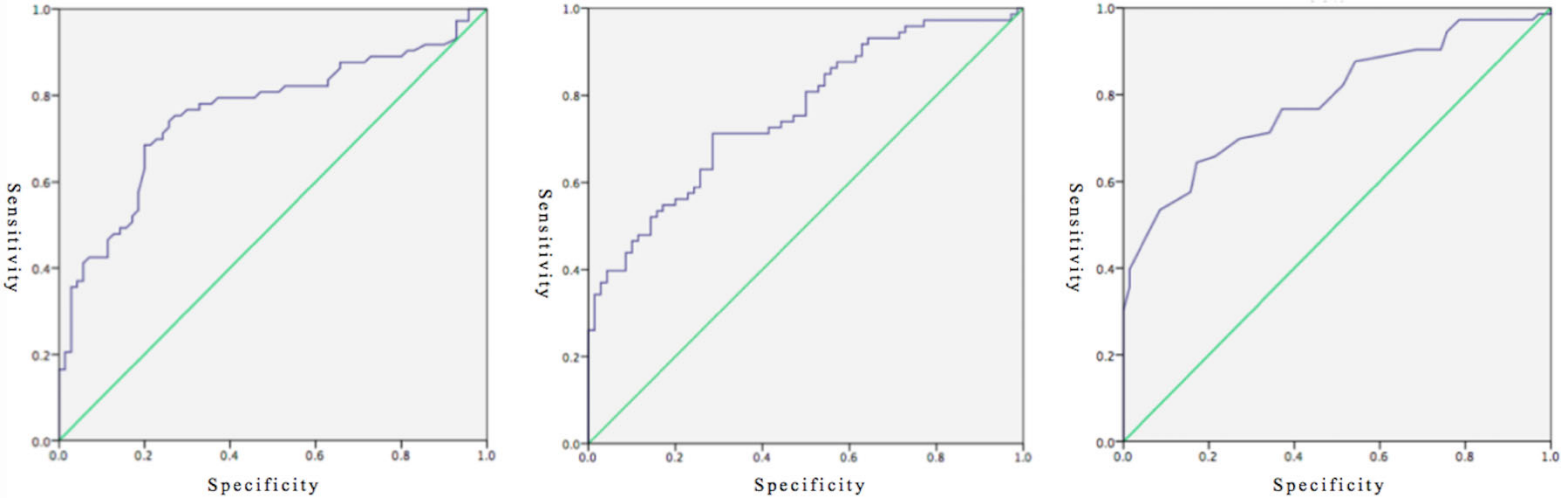
Receiver operator characteristic curves of SI‐A1, SI‐B2, and SI‐C1.

Among the six indicators, the areas under the curve of SI‐A2 and SI‐B1 were 0.66, while those of the other indicators were all above 0.7, reaching a moderate diagnostic accuracy. The sensitivity of SI‐B1 was low; that is, it could not screen intellectual disability well, but the specificity was high, meaning that it could exclude the normal population extremely well. This result might be because the task was relatively simple, and the degree of differentiation was relatively low. In general, SI‐A1, SI‐B2, and SI‐C1 were better identification indices of intellectual disability, and the ROC curves of the three are shown in Figure 1.

### User experience analysis

A chi‐square test was conducted on the number of “likes”, “neutral”, and “dislikes” of SI tasks and traditional paper‐and‐pencil tasks. There was a significant difference between the two types of tasks (*χ* ^2^ = 24.87, *p* < .001). Children preferred SI tasks to traditional tasks. There were 96 children (80.0%) willing to perform the SI tasks again and only 69 (57.5%) willing to perform the traditional tasks again. There was a significant difference between the two proportions (*χ* ^2^ = 4.91, *p* = .027). A total of 83 children (69.2%) said they preferred SI tasks, 30 (25.0%) preferred traditional tasks, and 7 (5.8%) thought they were the same. The reasons why children liked a task mainly included feeling interested, acquiring skills, and achieving a sense of achievement, while children disliked a task mainly owing to boredom, difficulty, and nervousness. In short, children preferred SI tasks.

In order to ensure scoring reliability, the researchers randomly selected six videos (four from ID children) before formal scoring, and then two researchers scored the participants' engagement and emotional enthusiasm respectively. The consistency coefficient of engagement was 0.83, and the consistency of emotional enthusiasm was 1.00, indicating good interrater reliability. Then, the engagement and emotional enthusiasm of the SI tasks and traditional tasks were rated and compared.

The repeated measurement analysis of variance for the engagement of different tasks showed that there was a significant difference among tasks [*F* (1.91, 36.19) = 3.84, *p* = .033, *η* ^2^_*p*_  = 0.168]. The post‐test used Bonferroni correction. The engagement score for SI Coding (0.05 ± 0.05) was significantly lower than that for the traditional task (0.61 ± 0.28) (*p*s = .045). Engagement scores for SI Go‐Nogo (0.35 ± 0.18) and SI Stop‐Signal (0.55 ± 0.17) were lower than those for the traditional task, but the differences were not statistically significant (*p*s > .05). The lower the engagement score, the more focused the individual was; that is, the SI tasks could attract the individual's attention, and the individual was more involved in them. The analysis of emotional positivity showed that there was a difference among tasks [*F* (1.99, 37.74) = 7.67, *p* = .002, *η* ^2^ *_p_*  = 0.288]. The emotional enthusiasm for the SI Go‐Nogo (4.90 ± 0.07) and SI Coding (4.65 ± 0.15) tasks was significantly higher than that for the traditional tasks (4.14 ± 0.27) (*p*s < .05). Enthusiasm for the SI Stop‐Signal (4.30 ± 0.18) was higher than that for the traditional tasks, but the difference was not statistically significant (*p* = .419). In conclusion, children had higher engagement and more positive emotions when conducting SI tasks.

## DISCUSSION

### The SI assessment tool could effectively measure the executive function of children with ID

The six indices of the three SI tasks were significantly correlated with all the traditional indices, the correlation of most indices reached a medium to a high degree, and all the tasks had good criterion validity. The direction of the correlation coefficient revealed that children with higher SI‐A1, SI‐B1, and SI‐C1 scores had better inhibition performance, higher cognitive flexibility, faster processing speed, and greater memory capacity on traditional measurement tasks. The longer the response time in the SI tasks was, the weaker the inhibition function, the lower the cognitive flexibility, the slower the cognitive processing speed, and the smaller the memory capacity.

There were differences in the content of executive function measured by the three SI tasks. According to the correlation coefficient, in the SI Go‐Nogo task, SI‐A1 was moderately correlated with inhibition, cognitive flexibility, and processing speed, but was weakly correlated with memory capacity. SI‐A1 was a comprehensive index. SI‐A2 had a moderate correlation with cognitive processing speed but only a weak correlation with other traditionally measured indices. Response time was a single‐dimension index, which mainly reflected the processing speed. Considering that the correlation between SI‐A1 and traditional indices was stronger than that of SI‐A2, SI‐A1 was superior to SI‐A2 and could more comprehensively and accurately represent individual executive function. In the Stop‐Signal task, the correlation coefficients between SI‐B1, SI‐B2, and each criterion had no significant difference in numerical value, and they were all good characterization indices. In the SI Coding task, SI‐C1 and SI‐C2 had a medium to a high degree of correlation with the criteria. SI‐C1 and SI‐C2 were comprehensive indices that represented many aspects of executive function. The two indices of the coding task better represented processing speed and inhibition than memory capacity. SI‐C1 and SI‐C2 had a similar quality of representation, and both were comprehensive indices. SI Coding was the best tool to measure ID children's execution function, followed by SI Go‐Nogo and SI Stop‐Signal. Different tasks or indices could be selected in follow‐up research according to different research intentions.

All three SI tasks can effectively distinguish TD children from ID children, and the executive function of ID children is significantly weaker than that of TD children. Previous studies generally agree that children with ID have certain executive dysfunction. For example, Memisevic and Sinanovic (2014) and Shishido et al. (2020) supported this conclusion using the executive functional behavior rating scale. Danielsson et al. (2010) used behavioral measures to indicate that adults with ID had executive function problems. Danielsson et al. (2012) used behavioral measures to indicate that children with ID had weaker executive functions than TD children, for example regarding inhibition and nonverbal working memory capacity. Memisevic and Sinanovic's (2014) study showed that the degree of ID affected executive function, while Putko et al. (2017) showed that children with moderate ID had significant executive dysfunction and children with mild ID did not. The inconsistencies in these findings suggest that the measurement methods and indices of executive function differed in their ability to distinguish the level of ID. In this study, SI‐A1, SI‐C1, and SI‐C2 could effectively distinguish children with mild and moderate ID, while the other three indices could not effectively distinguish the degree of ID.

In conclusion, the three SI tasks could effectively measure children's executive function and had good criterion validity and discrimination. SI‐A1 could represent inhibition function, processing speed and cognitive flexibility. SI‐A2 specifically represented processing speed and could distinguish the degree of ID. SI‐B1 and SI‐B2, especially SI‐B1, could reflect processing speed, cognitive flexibility and inhibition level. SI‐C1 and SI‐C2 are comprehensive indices that could reflect children's executive function relatively comprehensively and distinguish the degree of ID.

### The SI assessment tool had good sensitivity and specificity

The ROC is a good tool to evaluate the differential diagnosis ability of a continuous index (Hanley & Mcneil, 1982; Mahdizadeh & Zamanzade, 2021). The area under the curve (AUC) represents the diagnostic accuracy, and sensitivity and specificity represent the probability of missed diagnosis and misdiagnosis (Metz, 1978; Pencina et al., 2015).

The AUC of SI‐A1 was greater than 0.7, indicating moderate accuracy as a diagnostic index for children with ID. The AUC of SI‐A2 was slightly less than 0.7 and had low accuracy. The sensitivity of the two indices was low, the specificity was good, and the diagnostic coincidence rate was medium. If the diagnosis is based on the performance of the SI Go‐Nogo task, a high level of missed diagnosis and a low level of misdiagnosis might occur, and the sensitivity needs to be improved. The AUC of SI‐B1 was 0.66, slightly lower than 0.7, indicating low accuracy as a diagnostic index for children with ID. The AUC of SI‐B2 was higher than 0.7 with moderate accuracy. SI‐B1 had low sensitivity, high specificity, and a low diagnostic coincidence rate, while SI‐B2 had medium sensitivity, specificity and diagnostic coincidence rate. If a diagnosis is made based on SI‐B1, there might be a high rate of missed diagnosis, and the sensitivity needs to be improved. SI‐B2 was balanced in all aspects. Both Si‐C1 and SI‐C2 had an AUC of 0.79. In the diagnosis of children with ID, the accuracy was moderate, the sensitivity was low, the specificity was high, and the diagnosis coincidence rate was medium. The sensitivity and specificity were better than those of the other tasks.

Most indices of the three SI tasks had moderate accuracy in ID diagnosis. The missed diagnosis rate of the three tasks was generally high, and the misdiagnosis rate was generally low. In other words, these tasks were not sensitive to intellectual disabilities, but their reliability was high once a diagnosis was made. Missed diagnoses in children with ID may affect the efficacy of subsequent interventions. Therefore, further research is needed to improve the sensitivity of these tasks. Although this SI tool has a certain diagnostic accuracy, it cannot be used as a separate assessment tool at present and should be combined with other tools for comprehensive judgement. When applied in later research or practice, the indices should be reasonably used according to the research needs. For an accurate diagnosis, other tools with high sensitivity should be used.

### The SI assessment tool had better user experience and higher user engagement

The children had a higher preference for SI tasks than for traditional tasks. In SI tasks, children had a higher level of engagement and showed more positive emotions. This result might be influenced by the use of sports games. Previous studies have shown that assessment in a game environment can make young students more engaged and motivated than they are in traditional assessment tasks that encourage students to reach their full potential (Perrotta et al., 2013). When the assessment task was presented in the form of a game, the player (the assessed person) could participate in the task with high attention and for a long time without any external pressure (Hautala et al., 2020). On the other hand, this result might be related to the developmental characteristics of the participants. In this study, TD children and ID children were recruited from primary and middle schools, with an average age of approximately 12 years old. They preferred games or sports. The traditional measure of executive function was the pen‐and‐paper test, which was a static measurement, while the SI game, which required the full participation of the individual, was a dynamic measurement. Third, SI tasks could better engage the children's initiative and autonomy. In the traditional tasks, children passively completed the experimenter's requirements, and the experimenter was the leader; thus, this process was relatively boring. However, the operation interface was more attractive in SI tasks, and the testing process was more interesting. The testing process required body movements, and the interactive operation made children feel autonomous (Don & O'Byrne, 2022). These factors might lead children to have a greater preference for SI tasks and to be more engaged in the assessment process and to experience more positive emotions.

Academic evaluation of tools pays more attention to the hard power of tools, such as the test of reliability and validity, but often ignores soft power, such as the subjective feelings and cooperation willingness of individuals in the measurement process. If the SI task is to be applied in the assessment of children with ID, it is necessary to consider the user experience of children. Owing to the developmental characteristics of children with ID, such as weak self‐control and attention that is easily distracted, measurement tools need good reliability and validity and children's willingness to participate for long periods to complete the test. Traditional assessment tools are more likely to make children feel tired and distracted, which will affect children's task performance and lead to an underestimation of ID. SI tasks reflect children's subjectivity, attract their attention, engage them more (Huizenga et al., 2009), and improve their task performance (Don & O'Byrne, 2022; Tangkui & Tan, 2020). Therefore, SI assessment tools can optimize and improve traditional tools, reflecting the development requirements of the era of product design (Ding et al., 2014) and the core value requirements of people‐orientation, thereby achieving a more accurate assessment of children's development.

## RESEARCH DEFICIENCIES AND PROSPECTS

The proposed embodied SI assessment tool is not a subversion of the traditional method but a supplement to the existing method. The embodied assessment tool based on SI technology has higher ecological validity, and the assessment content is more real and reliable. The tool can automatically collect comprehensive and detailed action information and quickly conduct efficient statistical analyses. The embodied assessment tool has higher user engagement and readily obtains the children's cooperation. This tool can optimize the existing assessment system.

This study also has some shortcomings. Although executive function is an important part of intelligence, intelligence also contains other rich content, such as language understanding, expression, and abstract reasoning. The assessment of children with ID should be systematic. In addition to intelligence tests, diagnostic criteria for mental disabilities proposed by the American Association for Intellectual Disabilities (AAIDD), World Health Organization (WHO) and American Psychiatric Association (APA) require tests constructed from sociological and anthropological perspectives. Emphasis is placed on the adaptive behaviours that individuals exhibit in their interactions with the environment and on the functional assessment of the support systems that individuals need for development (Luckasson & Schalock, 2013; Schalock et al., 2018). Therefore, the SI tool designed in this study is only one part of the assessment system of children with ID and cannot be used as a diagnostic tool on its own. The tasks adopted in this study mainly measure executive function. More systematic studies are needed to realize the embodied assessment of children with ID and to apply SI technology to the assessment field. In addition, these assessment tools are based on SI devices that are costly to use and maintain, which may pose challenges for widespread implementation, particularly in resource‐constrained settings such as developing countries. Reducing the cost of these tools or finding alternative cost‐effective devices may help solve these problems.

The design of the embodied assessment tool based on SI technology is an application exploration of technologies and theories in diagnosing children with ID. These technologies and theories are helpful for assessing cognitive ability and can also be used to assess social skills and interpersonal emotions. The high ecological validity characteristics of the assessment scene make the results more real and comprehensive. These assessment tools can be used in scenarios or institutions that require the assessment of children with ID. For example, in rehabilitation centers, these tools can be used for rapid assessment before and after rehabilitation training for children with ID, which enables timely identification of issues, adjustment of rehabilitation plans, and ensures that children receive optimal treatment and support. These tools can be used in schools or educational institutions as part of the learning and educational support of children with ID, thereby providing them with personalized learning experiences and teaching content. These tools can also be used for integration with intervention tools for children with ID. SI technology is of great value in interventions for children with ID. Some scholars have tried to apply SI games to interventions for children with special needs and achieved good intervention effects. For example, Xu and Zhu (2016) showed that SI games could effectively promote the physiological function and other abilities of children with ID. The integration of SI assessment tools and digital rehabilitation training tools will greatly improve the efficiency of intervention for children with ID.

## CONFLICT OF INTEREST STATEMENT

On behalf of all authors, the corresponding author states that there is no conflict of interest.

## ETHICS STATEMENT

Ethical approval was obtained prior to the study from the Human Research Ethics Committee of East China Normal University (China).

## Data Availability

The data used to support the findings of this study are available from the corresponding author upon request.
